# Regulating the Polypyrrole Ion-Selective Membrane and Au Solid Contact Layer to Improve the Performance of Nitrate All-Solid Ion-Selective Electrodes

**DOI:** 10.3390/mi14040855

**Published:** 2023-04-14

**Authors:** Weizhuo Gao, Weixuan Jing, Yanrui Du, Zehao Li, Pengcheng Liu, Feng Han, Libo Zhao, Zhaochu Yang, Zhuangde Jiang

**Affiliations:** 1 State Key Laboratory for Manufacturing Systems Engineering, Xi’an Jiaotong University, Xi’an 710049, China; 2 Chongqing Key Laboratory of Micro-Nano Systems and Smart Transduction, Chongqing Technology and Business University, Chongqing 400067, China; 3 Shandong Laboratory of Yantai Advanced Materials and Green Manufacturing, Yantai 265503, China

**Keywords:** all-solid ion-selective electrode, polypyrrole ion-selective membrane, Au solid contact layer, surface morphology, wettability

## Abstract

With polymerization duration and Au^3+^ concentration of the electrolyte regulated, a desirable nitrate-doped polypyrrole ion-selective membrane (PPy(NO_3_^−^)-ISM) and Au solid contact layer of anticipate surface morphology were obtained, and the performance of nitrate all-solid ion-selective electrodes (NS ISEs) was improved. It was found that the roughest PPy(NO_3_^−^)-ISM remarkably increases the actual contact surface area of the PPy(NO_3_^−^)-ISMs with nitrate solution, which leads to better adsorption of NO_3_^−^ ions upon the PPy(NO_3_^−^)-ISMs, and produces a larger number of electrons. The most hydrophobic Au solid contact layer avoids the formation of the aqueous layer at the interface between the PPy(NO_3_^−^)-ISM and Au solid contact layer, and ensures unimpeded transporting of the produced electrons. The PPy-Au-NS ISE for polymerization duration 1800 s and at Au^3+^ concentration 2.5 mM of the electrolyte displays an optimal nitrate potential response, including a Nernstian slope of 54.0 mV/dec, LOD of 1.1 × 10^−4^ M, rapid average response time less than 1.9 s, and long-term stability of more than 5 weeks. This indicates that the PPy-Au-NS ISE is an effective working electrode for the electrochemical determination of NO_3_^−^ concentration.

## 1. Introduction

Wastewater containing nitrates derived from the effluent of explosives, fertilizers, and drugs has become a worldwide issue due to its toxicity and carcinogenicity, and can lead to the enrichment of water [1,2,3]. Thus, NO_3_^−^ concentration determination involving spectrophotometry [4], chromatography [5], and potentiometric detection based on ion-selective electrodes (ISEs) [6,7,8] has been widely researched. In comparison with other methods, the latter has many advantages, such as simple instrumentation, rapid response time, portability, low energy consumption, separation-free, unaffected by color or turbidity, wide dynamic range, and reasonable selectivity [9,10,11]. The typical ISEs include ISEs with internal solution [12] and nitrate all-solid ISEs (NS ISEs) [13]. Compared with the former, NS ISEs are characterized with low-cost, easy fabrication, no contamination, and nondestructive testing [9,13]. A typical NS ISE is composed of an ion-selective membrane (ISM), intermediate solid contact layer, and substrate electrode. In order to improve the nitrate potential response of NS ISEs, ISMs with excellent ion selectivity and reasonably conductive intermediate solid contact layer have been constructed in the literature [13,14,15,16,17,18,19,20,21,22,23,24,25,26].

The electron- and ion-doped conducting polymers such as tetrathiafulvalene (TTF), poly-thiophene (PT), poly-(3-octylthiophene) (POT), and poly-aniline (PANI) have been broadly employed for the construction of ISMs [14,15,16,17]. For example, Alizadeh synthesized cyclo[4]pyrrole ether on a carbon paste electrode and created the chemically modified carbon paste electrode (CMCPE) for nitrate concentration detection; the CMCPE has a Nernstian slope of 57.5 mV/dec, limit of detection (LOD) of 10^−5^ M, and response time of less than 10 s [14]. Piek fabricated a NS ISE based on NO_3_^−^-doped TTF ISM for nitrate concentration detection with a Nernstian slope of 59.14 mV/dec and LOD of 10^−6.2^ M [15]. Moreover, due to its excellent NO_3_^−^ ion selectivity and easy film formation, the NO_3_^−^-doped polypyrrole ISM (PPy(NO_3_^−^)-ISMs) has been widely employed in NS ISEs [18,19,20]. For instance, Pu employed a PPy(NO_3_^−^)-ISM to fabricate the NS ISE, which exhibited a Nernstian slope of 55 mV/dec, LOD of 10^−5^ M, and response time of less than 20 s [18]. Zhang systemically investigated the NO_3_^−^ ion selectivity of the PPy(NO_3_^−^)-ISM, and found that the ISM shows excellent NO_3_^−^ ion selectivity, including selectivity coefficients (log *K_ij_*) for Cl^−^ of −3, HCO_3_^−^ of −3.5, H_2_PO_4_^−^ of −4.8, and SO_4_^2−^ of −4.9 [19]. Álvarez-Romero utilized NO_3_^−^ ion-doped polypyrrole as a recognition agent to build a novel NS ISE; this ISE exhibits a Nernstian slope of 57.1 mV/dec with a LOD of 5.37 × 10^−5^ M [20]. Although in the present literature, PPy(NO_3_^−^)-ISMs with good NO_3_^−^ ion selectivity are put forward, the random rough solid–liquid contact interface of the PPy(NO_3_^−^)-ISM with nitrate solution is frequently regarded as an ideal smooth one. The relationships among synthesizing parameters, surface morphology, wettability, and nitrate potential response of ISMs have not been established. This seriously hinders the performance improvement of the ISM.

Moreover, the formation of ISMs unavoidably introduces O_2_, CO_2_, and H_2_O, which further forms an aqueous water layer between the ISM and underlying substrate. This seriously reduces the nitrate potential response of the ISEs [13,21]. Thus, a desired intermediate solid contact layer also requires strong hydrophobicity [13,18,22,23]. Due to their extremely low surface free energy and strong conductivity, carbon-based materials (e.g., graphene (GR)) [13,18], nanostructured noble metals (e.g., Au) [22], and polymers (e.g., POT and PANI) [23] are frequently used in the literature. For instance, Tang formed a GR solid contact layer for a NS ISE, and gained a Nernstian slope of 57.9 mV/dec, LOD of 3 × 10^−5^ M, and response time within 10 s for NO_3_^−^ concentration detection [13]. Pu fabricated the PPy(NO_3_^−^) ISE with a GR solid contact layer, and detected the nitrate solution with a Nernstian slope of 56 mV/dec, LOD of 10^−5^ M, and response time of less than 10 s [18]. Chen prepared a PPy(NO_3_^−^) ISE with a nanohybrid composite film of gold nanoparticles and electrochemically reduced graphene oxide, and the PPy(NO_3_^−^) ISE exhibited a detection range of 10^−5^ to 10^−1^ M with a theoretical LOD of 10^−5.2^ M [22]. Khripoun synthesized NS ISEs with POT and PANI solid contact layers, which perform a nitrate potential response including a Nernstian slope of 53 mV/dec and LOD of 10^−5^ M [23]. Due to its great chemical stability, high hydrophobicity, and low electrical resistance, the Au layer has already been employed as an intermediate solid contact layer for K^+^, Na^+^, and Cu^2+^ all-solid ISEs [24,25,26]. For instance, Xu synthesized hydrophobic monolayer-protected gold nanoclusters (MPGNs) on a glassy carbon electrode (GCE) as a redox solid contact layer for K^+^-MCPs-ISEs, and the K^+^-MCPs-ISE had a Nernstian slope of 55.1 ± 1.22 mV/dec and LOD of 1.55 × 10^−7^ M [24]. By one-step electrodeposition, Wang fabricated a hydrophobic gold nanodendrite solid contact layer for the Na^+^ ISE, and gained a Nernstian slope of 56.58 ± 1.02 mV/dec with a LOD of 0.8 × 10^−6^ M [25]. Woz’nica formed a dithizone decorated gold nanoparticle solid contact layer for the Cu^2+^ ISE, and subsequently detected CuSO_4_ solutions with a Nernstian slope of 22.1 mV/dec and LOD of 10^−7.5^ M [26]. Although different Au solid contact layers have been employed to construct various ISEs, the effect of the surface morphology on the wettability of Au solid contact layer, and furthermore, the potential response of the ISEs, has been frequently neglected in the literature. This impedes the synthesizing parameter optimization of the Au solid contact layers and the performance improvement of relative ISEs.

In this paper, PPy-Au-NS ISEs were synthesized for different polymerization durations and at various Au^3+^ concentrations of the electrolyte. The relationship among the polymerization duration, the surface morphology, and the wettability of the PPy(NO_3_^−^)-ISM, as well as the NO_3_^−^ potential response of the PPy(NO_3_^−^)-ISM, was investigated. The effect of the Au^3+^ concentration of the electrolyte on the surface morphology and wettability of the Au solid contact layer was formulated. Subsequently, the polymerization duration and Au^3+^ concentration of the electrolyte were regulated, and the optimal NO_3_^−^ detection performance of the ISEs was obtained.

## 2. Experimental Details

### 2.1. Materials and Apparatus

Pyrrole monomer (Aladdin Industrial Corporation, Shanghai, China) and chloroauric acid (HAuCl_4_, Aladdin Industrial Corporation), sodium nitrate (NaNO_3_, Tianli Chemical Reagents Co. Ltd., Tianjin, China), sodium chloride (NaCl, Tianli Chemical Reagents Co. Ltd., Tianjin, China), potassium ferricyanide (K_3_[Fe(CN)_6_], Tianli Chemical Reagents Co. Ltd., Tianjin, China), absolute ethyl alcohol, and acetone are all analytical reagents. The glassy carbon electrode (GCE, 3 mm diameter), saturated calomel electrode, Ag/AgCl electrode, and platinum wire electrode were provided by Xuzhou Zhenghao Electronic Technology Co. Ltd. (Xuzhou, China). De-ionized (DI) water (R ≥ 18.25 MΩ·cm) was utilized.

A magnetic stirrer (BII-3, Shanghai Sile Automation Science & Technology Co. Ltd., Shanghai, China), ultrasonic washer (KQ-100DE, Kunshan Ultrasonic Instruments Co. Ltd., Kunshan, China), electronic balance (FA1004N, Changzhou Xingyun Electronic Equipment Co. Ltd., Changzhou, China), laser scanning confocal microscope (LSCM, LS4000, Olympus, Tokyo, Japan), scanning electron microscope (SEM, SU8010, Hitachi, Tokyo, Japan), atomic force microscope (AFM, INNOVA, Veeco, New York, NY, USA), contact angle (CA) meter (OCA20, Dataphysics Company, Filderstadt, Germany), and an electrochemical workstation (CHI660D, Shanghai Chenghua Instrument Co. Ltd., Shanghai, China) were employed.

### 2.2. Construction of the PPy-Au-NS ISEs

**Figure 1:**
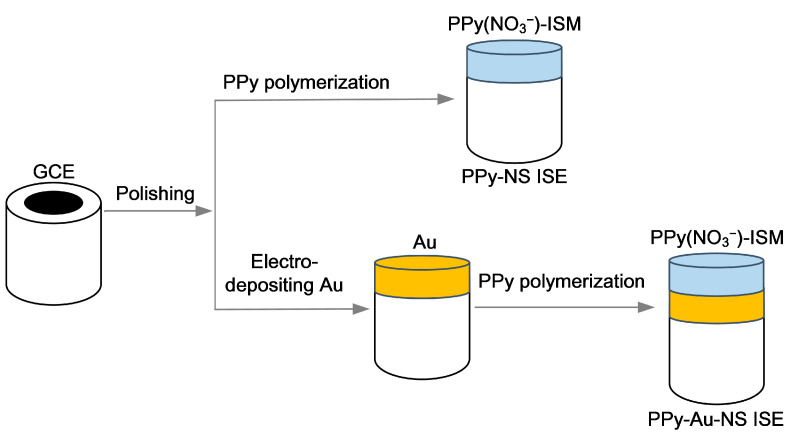
Synthesizing process for PPy-NS ISE and PPy-Au-NS ISE.

Figure 1 shows the synthesizing process for PPy-Au-NS ISEs. GCEs were polished using 0.3 and 0.05 μm aluminum oxide powder. Different nanostructured Au solid contact layers were electrodeposited onto the as-prepared GCEs with a cyclic voltage ranging from −1.4 V to 0.2 V, and the Au^3+^ concentration of the electrolyte was 1, 2, 2.5, 3, 4, and 5 Mm, respectively. Once coated with the Au solid contact layer, GCEs were immersed in a mixture of 0.5 M pyrrole and 10 mM NaNO_3_ solution, under a constant voltage 0.7 V, and the PPy(NO_3_^−^)-ISM was electrochemically polymerized onto the Au solid contact layer for a polymerization duration 1800 s. The electrochemical polymerization was performed with a three-electrode system, that is, the GCE coated with the Au solid contact layer as the working electrode, a saturated calomel electrode as the reference electrode, and a platinum wire as the counter electrode. Subsequently, the PPy-Au-NS ISEs were constructed. Similarly, to investigate the effect of the polymerization duration on the PPy(NO_3_^−^)-ISM, different PPy-NS ISEs were fabricated for polymerization durations of 600, 1200, 1800, 2400, 3000, and 3600 s, respectively, as demonstrated in Figure 1.

### 2.3. Characterization of the As-Prepared PPy (NO_3_^−^)-ISMs and Au Solid Contact Layers

Surface morphologies of PPy (NO_3_^−^)-ISMs on the GCE were quantitatively characterized by LSCM, and the roughness *Sa*, correlation length *Sal*, skewness *Ssk*, as well as kurtosis *Sku* were obtained. Au solid contact layers were characterized by SEM at 3 and 5 kV, and the maximum height *Sz*, mean square height *Sq*, *Ssk*, and *Sku* of Au solid contact layers were acquired by AFM. Furthermore, with a water droplet of 5 μL in contact with both PPy (NO_3_^−^)-ISMs and Au solid contact layers, the CA values of the PPy (NO_3_^−^)-ISMs and Au solid contact layers were measured using a CA meter, respectively.

### 2.4. Electrochemical Characterization of the PPy-Au-NS ISEs

Electrochemical characterizations of the PPy-Au-NS ISEs were all performed with a three-electrode system, that is, PPy-Au-NS ISEs as the working electrode, the Ag/AgCl electrode as the reference electrode, and a platinum wire as the counter electrode. Electrochemical impedance spectra (EIS) of the PPy-Au-NS ISEs were tested in 0.1 M NaNO_3_ solution at the open-circuit potential within the frequency range of 10 kHz to 0.1 Hz. Electromotive force (EMF) curves of different PPy-Au-NS ISEs were measured at NO_3_^−^ concentrations of 10^−1^, 10^−2^, 10^−3^, 10^−4^, 10^−5^, and 10^−6^ M of NaNO_3_ solution; each EMF curve was determined by five working electrodes synthesized using the same parameters. Likewise, EIS and EMF curves of different PPy-NS ISEs were also acquired. Moreover, water layer tests of both PPy-Au-NS ISE and PPy-NS ISE were conducted in an unstirred NaNO_3_ solution at room temperature. The redox sensitivity of the PPy-Au-NS ISE was measured in a solution of 1 mM total concentration of the Fe(CN)_6_^3−/4−^ redox couple, with the ratio of Fe(II)/Fe(III) ranging from 1/10 to 10/1 at a constant ionic background of 0.1 M NaNO_3_. Via the separate solution method, the ion selectivity of the as-prepared PPy-Au-NS ISE was tested in 0.01 M NaNO_3_, NaCl, Na_2_SO_4_, Na_2_HPO_4_, and NaI solutions, and the selectivity coefficients (log *K_ij_*) were determined.

## 3. Results and Discussion

### 3.1. Different PPy(NO_3_^−^)-ISMs on the GCE for Various Polymerization Durations

**Figure 2:**
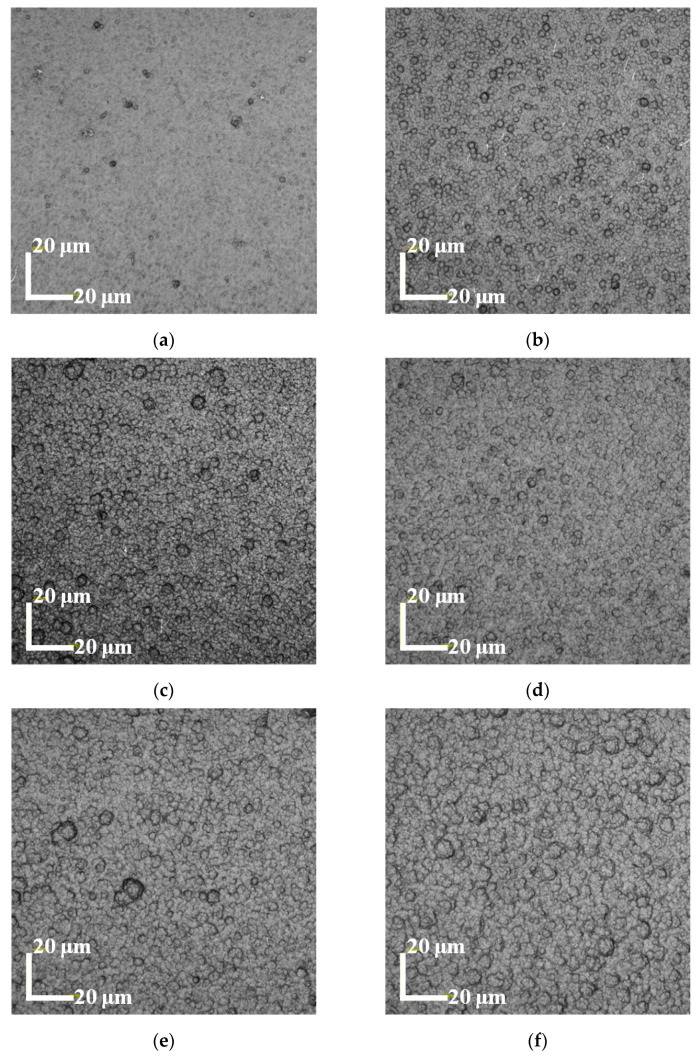
LSCM micrographs of the PPy(NO_3_^−^)-ISMs for polymerization durations 600 (**a**), 1200 (**b**), 1800 (**c**), 2400 (**d**), 3000 (**e**), and 3600 s (**f**).

Figure 2 shows that all PPy(NO_3_^−^)-ISMs under different polymerization durations completely cover the top surface of GCE. More importantly, with the polymerization duration increasing, the diameters of the nodular structures on the PPy(NO_3_^−^)-ISM enlarge steadily (referring to Figure 2a–f), while the number of the nodular structures increases first (Figure 2a–c), then decreases significantly (Figure 2d), and subsequently increases again (Figure 2e,f). This is caused by both the vertical and horizontal growth of the PPy(NO_3_^−^)-ISM, that is, the increasing polymerization duration gives rise to both larger nodular structures on the PPy(NO_3_^−^)-ISM (vertical growth) and more side-by-side coalescence of neighboring nodular structures (horizontal growth). This suggests that the polymerization duration significantly influences the surface morphology of the PPy(NO_3_^−^)-ISM. It is worth noting that the highest number of nodular structures on the PPy(NO_3_^−^)-ISM occurs when the polymerization duration is 1800 s, as shown in Figure 2c.

**Figure 3:**
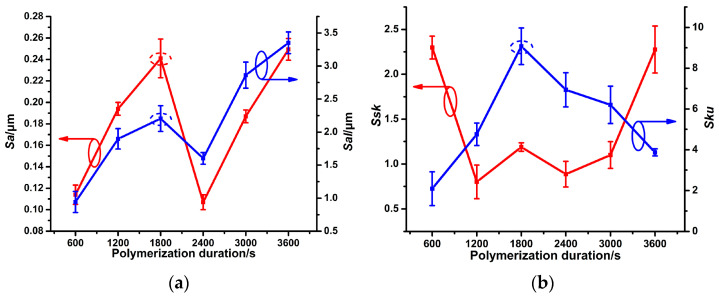
Effect of the polymerization duration on *Sa* and *Sal* (**a**), as well as *Ssk* and *Sku* (**b**), of the PPy(NO_3_^−^)-ISMs.

Figure 3a demonstrates that both *Sa* and *Sal* values of PPy(NO_3_^−^)-ISMs increase first, then decrease, and finally increase again. This is due to the variations in the diameters and number of nodular structures on the PPy(NO_3_^−^)-ISM, as presented in Figure 2a–f. The PPy(NO_3_^−^)-ISM for the polymerization duration 1800 s has the largest *Sa* value because it has the largest number of nodular structures (Figure 2c). This suggests that the surface of PPy(NO_3_^−^)-ISM for 1800 s is the roughest. Figure 3b shows that the *Ssk* value of PPy(NO_3_^−^)-ISM for the polymerization duration 1800 s is larger than those of durations 1200 and 2400 s (referring to Figure 2b–d). This means that sharper hills occur on the former PPy(NO_3_^−^)-ISM than on the latter ones. Furthermore, Figure 3b also shows that the *Sku* value of the PPy(NO_3_^−^)-ISM for polymerization duration 1800 s is the largest. This suggests that the surface of the PPy(NO_3_^−^)-ISM for polymerization duration 1800 s fluctuates most significantly, as demonstrated in Figure 2c. These findings indicate that the PPy(NO_3_^−^)-ISM for polymerization duration 1800 s has the roughest surface [27].

**Figure 4:**
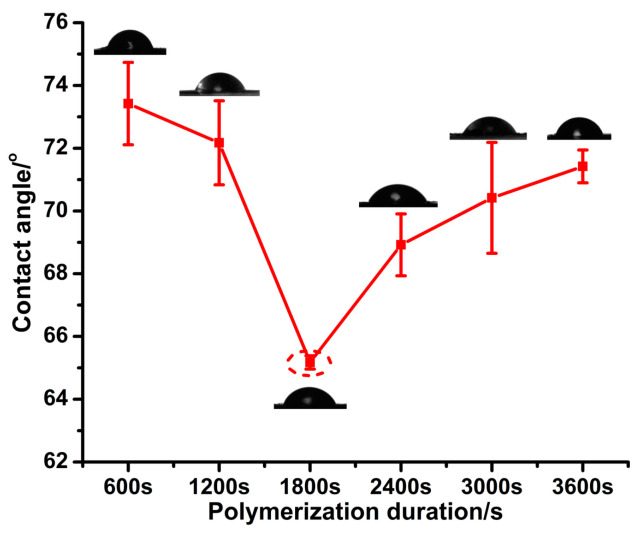
Effect of the polymerization duration on the CA values of the PPy(NO_3_^−^)-ISMs.

Figure 4 shows that the CA values of PPy(NO_3_^−^)-ISMs are all less than 90°, which means that the PPy(NO_3_^−^)-ISMs are hydrophilic. Furthermore, with the polymerization duration increasing, the CA value decreases first and then increases, and the smallest CA value corresponds to the polymerization duration 1800 s. This suggests that the roughest PPy(NO_3_^−^)-ISM has a most hydrophilic surface, as shown in Figure 3 and Figure 4. These findings suggest that the wettability of PPy(NO_3_^−^)-ISM belongs to the Wenzel state (the wetting state at which DI water is in complete contact with the whole rough surface), as described by Equation (1) [28,29],

cos *θ* = *r* cos *θ_e_* (1)

where *θ* is the contact angle of a water droplet upon a rough solid surface, *θ_e_* is the intrinsic contact angle of the specific material, and *r* is the non-dimensional surface roughness factor, which equals the ratio of the actual surface area to its flat projected area. With the polymerization duration increasing, the *Sa* and *Sku* values in Figure 3a,b suggest that the PPy(NO_3_^−^)-ISMs are rougher first, then become smoother, and subsequently rougher again, whilst the surface of the PPy(NO_3_^−^)-ISM fluctuates remarkably first, and then relatively gently. This makes the solid–liquid contact surface area of PPy(NO_3_^−^)-ISMs with DI water increase first and then decrease, that is, the *r* value in the Wenzel model enlarges first and then diminishes. This gives rise to the variation in the CA values displayed in Figure 4. The largest solid–liquid contact surface area of the PPy(NO_3_^−^)-ISM with DI water corresponds to 1800 s, which leads to the largest *r* value, and further gives rise to the smallest *θ*.

### 3.2. Various Au Solid Contact Layers at Different Au^3+^ Concentrations of the Electrolyte

**Figure 5:**
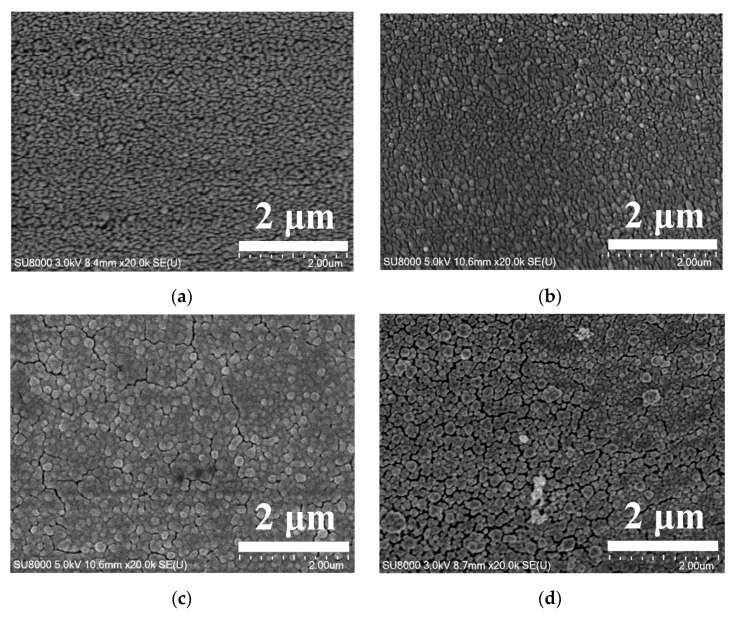

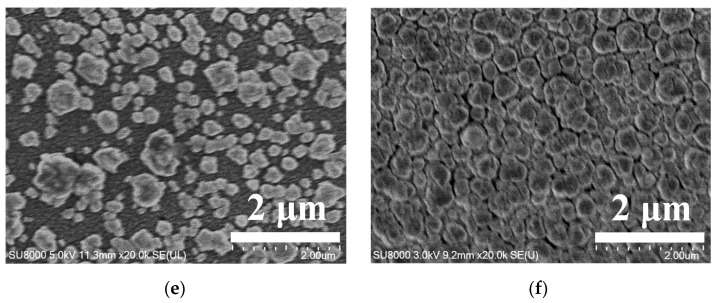
SEM micrographs of Au solid contact layers at Au^3+^ concentrations of 1 mM (**a**), 2 mM (**b**), 2.5 mM (**c**), 3 mM (**d**), 4 mM (**e**), and 5 mM (**f**) of the electrolyte, respectively.

Figure 5 shows that with the Au^3+^ concentration of the electrolyte increasing, Au nanoclusters on the Au solid contact layer enlarge gradually (Figure 5a–c), and then coalescence of adjacent Au nanoclusters occurs (Figure 5d–f). This gives rise to the fluctuation in the sizes of the gaps among neighboring Au nanoclusters, generally decreasing first and then increasing remarkably. All these responses are attributed to the increasing concentration of Au^3+^ ions in diffusion zones on the GCE, which causes larger Au nanoclusters, and furthermore, gives rise to increased coalescence of adjacent Au nanoclusters. This indicates that the Au^3+^ concentration of the electrolyte apparently affects the surface morphologies of Au solid contact layers. Moreover, at the Au^3+^ concentration of 2.5 mM of the electrolyte, the Au nanoclusters are the most homogeneous in diameter (Figure 5c).

**Figure 6:**
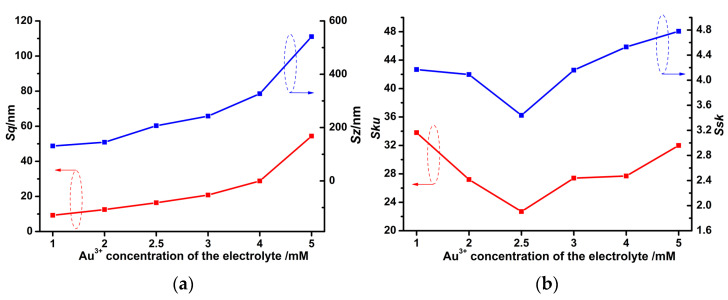
Effect of the Au^3+^ concentration of the electrolyte on *Sz* and *Sq* (**a**), as well as *Ssk* and *Sku* (**b**), of Au solid contact layers.

Figure 6a demonstrates that with the Au^3+^ concentration of the electrolyte increasing, both *Sz* and *Sq* curves climb monotonically. This is due to the larger Au nanoclusters on the Au solid contact layers (Figure 5a–f). Figure 6b shows that the *Ssk* and *Sku* values of all Au solid contact layers are larger than 0 and 3 (values of the Gaussian random surface), which means more wide pits and sharp hills occur on the Au solid contact layers than that on the Gaussian random surface [27,30]. Attention should be given to the fact that the *Ssk* and *Sku* values of the Au solid contact layer in Figure 5c are the smallest compared to the other values in Figure 5. This suggests that the homogeneous diameters of the Au nanoclusters lead to the least fluctuation in surface heights, which results in both sufficient hills and adequate pits simultaneously occurring on the Au solid contact layer at Au^3+^ concentration 2.5 mM of the electrolyte [30], as can be seen in Figure 5c and Figure 6b.

**Figure 7:**
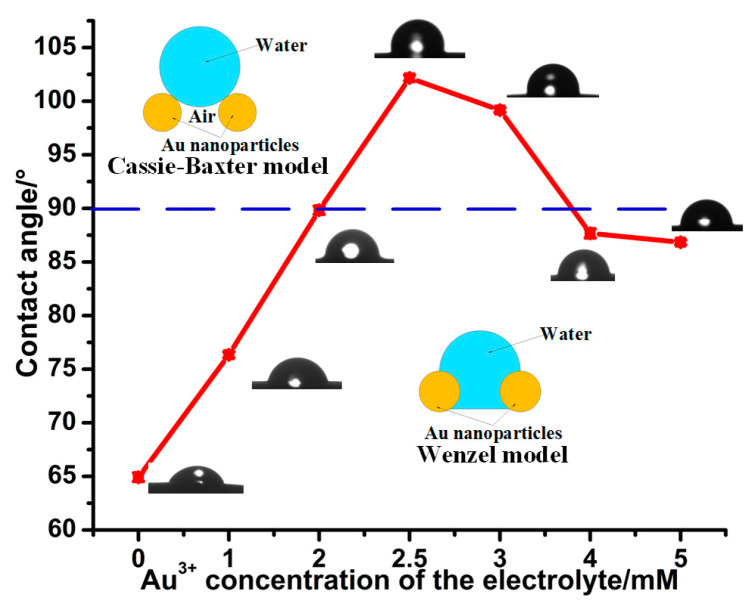
Effect of the Au^3+^ concentration of the electrolyte on the CA values of Au solid contact layers.

Figure 7 shows that the CA values of all Au solid contact layers are larger than those of pristine GCEs. With the Au^3+^ concentration of the electrolyte increasing, the CA values of Au solid contact layers increase first then decrease. The largest CA value of 103° corresponds to the Au^3+^ concentration of 2.5 mM of the electrolyte. This is attributed to the wettability of the Au solid contact layers being transferred from the Wenzel [29] to Cassie–Baxter state [31], as demonstrated in Figure 7. The Cassie–Baxter state (the wetting state at which DI water only touches the partial nanostructured rough surface due to air bubbles trapped in pockets [28,31]) is expressed as Equation (2),

cos *θ* = *f*_1_ cos *θ*_1_ + *f*_2_ cos *θ*_2_ (2)

where *θ*_1_ and *θ*_2_ are the solid–liquid and liquid–air contact angles, respectively, *f*_1_ and *f*_2_ are the solid–liquid and liquid–air area fractions, respectively, and *f*_1_ + *f*_2_ = 1. According to Equation (2), it is obvious that both sufficient hills and adequate pits on the Au solid contact layer at the Au^3+^ concentration of 2.5 mM of the electrolyte cause more air to be trapped in the pits among the hills, and thus results in the most stable Cassie–Baxter state, which further gives rise to the largest CA value (Figure 5c, Figure 6b and Figure 7).

### 3.3. Effect of Polymerization Durations on the NO_3_^−^ Potential Response of the PPy(NO_3_^−^)-ISMs

**Figure 8:**
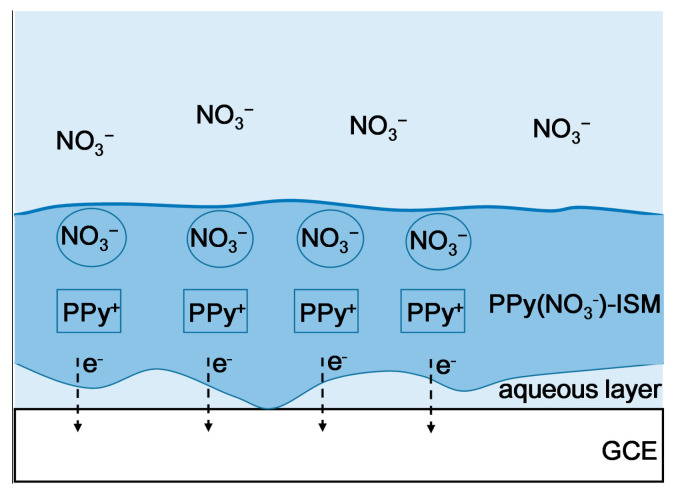
Schematic diagram of the NO_3_^−^ response procedure for the PPy-NS ISE.

Figure 8 illustrates that NO_3_^−^ ions are diffused from the nitrate solution upon the solid–liquid contact interface of the PPy(NO_3_^−^)-ISM with the nitrate solution (thick blue line in Figure 8). Then, these NO_3_^−^ ions are electrostatically attracted and trapped by the pores that are complementary to the size of NO_3_^−^ ions in the PPy(NO_3_^−^)-ISM. To counter the captured NO_3_^−^ ions, the PPy species transfer to the oxidation state PPy^+^, and the PPy(NO_3_^−^)-ISM produces the electrons [18,19]. These electrons are transported through the PPy(NO_3_^−^)-ISM and are gained by the GCE; furthermore, the NO_3_^−^ potential response is generated. However, the generated electron transmission would be seriously interfered with by the aqueous water layer between the PPy(NO_3_^−^)-ISM and GCE, as referred to in Figure 8.

**Figure 9:**
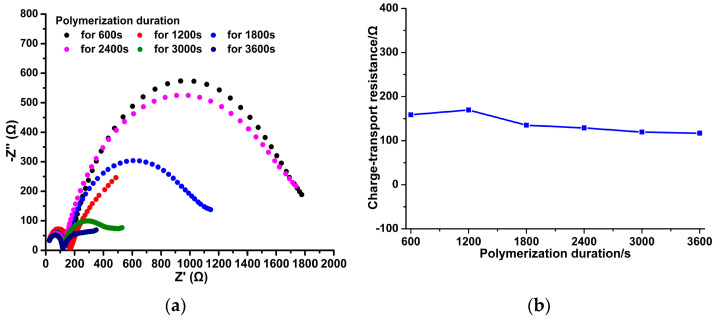
EIS with the equivalent circuit (**a**), and charge transfer resistance *Rct* (**b**) of PPy-NS ISEs in 0.1 M NaNO_3_ solution.

Figure 9a shows that for all EIS of PPy-NS ISEs, a semicircle exists at a low-frequency region. This corresponds to charge transporting resistances, *Rct*, together with interfacial diffusion impedances, *Zw*, in parallel with geometric capacitances, *Cd*, of the PPy(NO_3_^−^)-ISM in the equivalent circuit. As shown in Figure 9b, for all PPy(NO_3_^−^)-ISMs, the values of the *Rct* are less than 200 Ω and fluctuate slightly. The low *Rct* values represent that plenty of PPy species were oxidized to PPy^+^, while the produced electrons were transported directly through the PPy(NO_3_^−^)-ISM. Moreover, large *Cd* values (which are inversely proportional to *Rct* [32]) also indicate the sufficient oxidation of PPy species and strong conductivity of the PPy(NO_3_^−^)-ISM. Hence the solid–liquid contact surface area of the PPy(NO_3_^−^)-ISM with the nitrate solution significantly affects both the electrostatic adsorption of NO_3_^−^ ions and the electron production, and further influences the NO_3_^−^ potential response. Figure 9a also shows that a capacitive loop appears at the high-frequency region of the EIS, which represents the charge transporting resistance, *Rct_al_*, in parallel with the limited diffusion capacitance, *Cd_al_*, of the aqueous layer in the equivalent circuit [33]. A much larger radius of the high-frequency capacitive loop than that of the low-frequency one means a larger impedance and lower diffusion capacitance of the aqueous layer [32], which seriously hinders the electron transmission.

**Figure 10:**
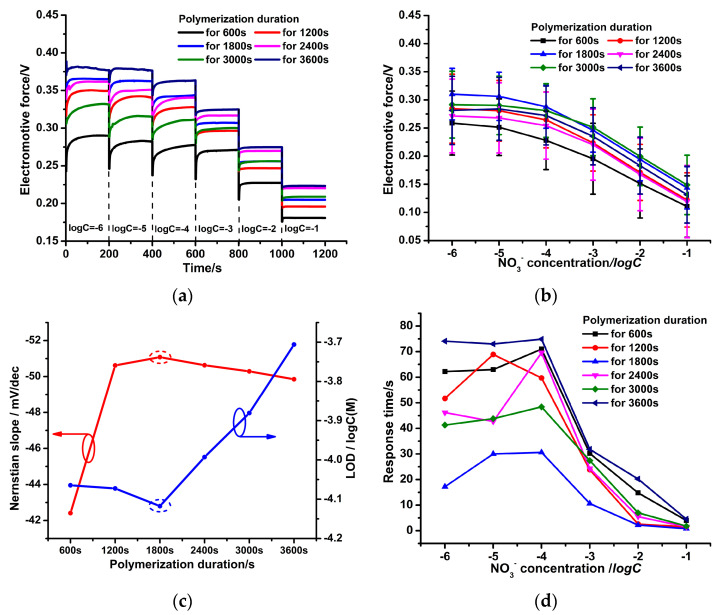
Time-dependent potential response traces of 6 freshly prepared PPy-NS ISEs (**a**). EMF curves of 6 freshly prepared PPy-NS ISEs, each curve was measured by 5 electrodes synthesized at the same parameters (**b**). Nernstian slope and low detection limit (**c**) as well as average response time (**d**) dependence on the logarithm of the activity of the PPy-NS ISE.

Figure 10a shows that when NO_3_^−^ concentration is below 10^−5^ M, the PPy-NS ISEs are insensitive to the change in NO_3_^−^ concentration. At high NO_3_^−^ concentration (above 10^−5^ M), the potential response of the PPy-NS ISEs on the NO_3_^−^ ions is stable, and the response time decreases remarkably with the increasing NO_3_^−^ concentration. This is because the larger NO_3_^−^ concentration enhances the mass transporting and adsorption of NO_3_^−^ ions on the PPy(NO_3_^−^)-ISM. Furthermore, EMF curves of six PPy-NS ISEs for different polymerization durations were obtained; each curve was measured using five electrodes synthesized at the same parameters for the EMF standard deviations, as shown in Figure 10b. Figure 10b reveals that for each PPy-NS ISE, the EMF standard deviations are large, which means that the performance of the PPy-NS ISE is unstable. This is due to the formation of the aqueous layer between the PPy(NO_3_^−^)-ISM and GCE, which seriously hinders the generated electron transmission, as referred to in Figure 8 and Figure 9a. Moreover, on the basis of the average values of EMFs in Figure 10b, the Nernstian slopes and LODs of the PPy-NS ISEs were determined (Figure 10c). Figure 10b–d demonstrates that the most hydrophilic PPy(NO_3_^−^)-ISM for polymerization duration 1800 s has the most stable EMF (with a standard deviation of 37.59–45.72 mV), the largest Nernstian slope of 51.08 ± 1.56 mV/dec (with linear correlation coefficient *R^2^* 0.9969), the lowest LOD of 8×10^−5^ mol/L, and the most rapid average response time of about 15.2 s. This is attributed to the largest solid–liquid contact surface area of the PPy(NO_3_^−^)-ISM with nitrate solution, which ensures the sufficient adsorption of NO_3_^−^ ions upon the PPy(NO_3_^−^)-ISM [34], and furthermore, generates adequate electrons. However, due to the interference of the aqueous layer between the PPy(NO_3_^−^)-ISM and GCE, the electrochemical performance the of PPy-NS ISEs is moderate regarding effective NO_3_^−^ concentration detection.

### 3.4. Effect of Au^3+^ Concentrations of the Electrolyte on the Electrochemical Performance of the PPy-Au-NS ISEs

**Figure 11:**
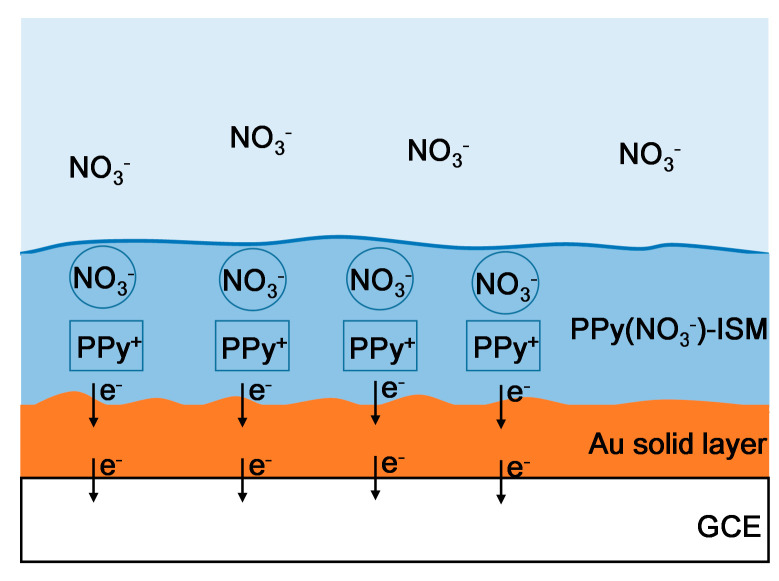
Schematic diagram of the NO_3_^−^ response procedure for the PPy-Au-NS ISE.

Figure 11 shows that the process of capturing NO_3_^−^ ions and producing electrons in the PPy(NO_3_^−^)-ISM of the PPy-Au-NS ISE is the same as that shown in Figure 8. However, due to the hydrophobicity of the Au solid contact layer, the formation of the aqueous layer is prevented, hence, the electrons generated in the PPy(NO_3_^−^)-ISM are transported directly through the Au solid contact layer to the GCE (Figure 11).

**Figure 12:**
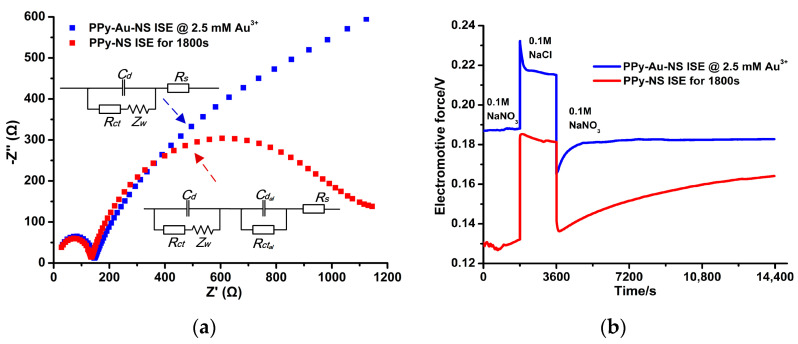
EIS with the equivalent circuit of PPy Au-NS ISE (**a**) and aqueous layer tests (**b**) of the PPy-NS ISE and PPy-Au-NS ISE in 0.1 M NaNO_3_ solution.

Figure 12a demonstrates that at the low-frequency region of the EIS, the radius of the semicircle of the PPy-Au-NS ISE is equal to that of the PPy-NS ISE. This suggests that the *Rct* of the PPy-Au-NS ISE is similar as that of the PPy-NS ISE, which is in reference to the equivalent circuit in Figure 12a. However, at the high-frequency region, the EIS of the PPy-Au-NS ISE is approximately linear, which represents the unlimited interfacial diffusion between the PPy(NO_3_^−^)-ISM and nitrate solution [13]. This suggests that extremely low charge transporting resistances exist between the PPy(NO_3_^−^)-ISM and GCE, that is, the Au solid contact layer excellently covers the surface of GCE, while the formation of the aqueous layer in Figure 8a is prevented in the PPy-Au-NS ISEs. This further allows the produced electrons to be easily transported from the PPy(NO_3_^−^)-ISM to the GCE, as referred to in Figure 11. Figure 12b shows that for the potentiometric response of the PPy-NS ISE, with the change from the interfering ion (Cl^−^) to the primary ion (NO_3_^−^), an obvious potential drift occurs. This suggests that an aqueous layer is formed in the PPy-NS ISE [13]. On the contrary, PPy-Au-NS ISE has a stable behavior, and reaches the potentiometric equilibrium rapidly after the alteration. This clearly indicates that the hydrophobic Au solid contact layer leads to the removal of the aqueous layer in the PPy-Au-NS ISEs [13].

**Figure 13:**
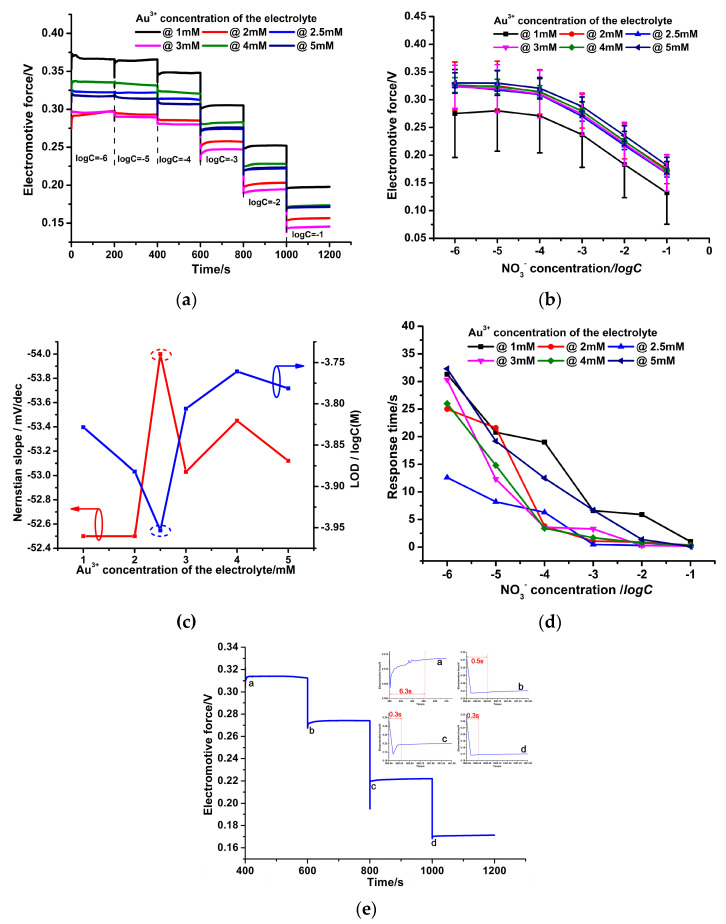
Time-dependent potential response traces of 6 freshly prepared PPy-Au-NS ISEs (**a**). EMF curves of 6 freshly prepared PPy-Au-NS ISEs, each curve was measured by 5 electrodes synthesized at the same parameters (**b**). Nernstian slope and low detection limit (**c**) as well as average response time (**d**) dependence on the logarithm of the activity of PPy-Au-NS ISE. Detailed response time of PPy-Au-NS ISEs at Au^3+^ concentration 2.5 mM of the electrolyte (**e**).

Figure 13a shows that compared to the PPy-NS ISE in Figure 10a, the PPy-Au-NS ISEs have a more stable potential response to the NO_3_^−^ ions and more rapid response time at various NO_3_^−^ concentrations. Similarly, the EMF curves of six PPy-Au-NS ISEs at different Au^3+^ concentrations of the electrolyte were determined; each curve was also measured by five electrodes synthesized at the same parameters. It is obvious that the EMF standard deviations of PPy-Au-NS ISEs in Figure 13b are significantly smaller than those of PPy-NS ISEs in Figure 10b. This is attributed to the fact that the hydrophobic Au solid contact layers prevent the formation of the aqueous water layer. Thus, in the PPy-Au-NS ISEs, the produced electrons were easily transported from the PPy(NO_3_^−^)-ISM to the GCE (Figure 11), which further leads to better potential response performance of the PPy-Au-NS ISEs than that of the PPy-NS ISEs. Based on the average values of the EMFs in Figure 13b, the Nernstian slope and LOD of PPy-Au-NS ISE were acquired (Figure 13c). Figure 13b–d reveals that the PPy-Au-NS ISE at Au^3+^ concentration 2.5 Mm of the electrolyte has the smallest EMF standard deviation of 3.17–10.14 Mv, the largest Nernstian slope of 54.0 ± 0.64 Mv/dec (with linear correlation coefficient *R^2^* 0.9945), the lowest LOD of 1.1 × 10^−4^ mol/L, and the most rapid average response time of less than 1.9 s (the average value of the response time 6.3, 0.5, 0.3, and 0.3 s corresponding to NO_3_^−^ concentration 10^−4^, 10^−3^, 10^−2^, and 10^−1^ M, respectively (Figure 13e)). This suggests that at Au^3+^ concentration 2.5 Mm of the electrolyte, the Au solid contact layer with sufficient hills and pits is the most hydrophobic (Figure 6b and Figure 7) [30], and further prevents the formation of the aqueous layer in the PPy-Au-NS ISE. This gives rise to the fastest and steadiest NO_3_^−^ potential response performance and outstanding fabrication stability of the PPy-Au-NS ISE.

Table 1 shows that the Nernstian slope and LOD of the PPy-Au-NS ISE in this work are comparable to those in the literature, while the average response time is significantly more rapid. This means that the NS ISE in this work is more suitable to the real-time NO_3_^−^ detection on the premise of effective response. In order to decrease the average response time of the ISE, the doping NO_3_^−^ concentration for the PPy(NO_3_^−^)-ISM is deliberately reduced. However, the PPy-Au-NS ISE still retains excellent NO_3_^−^ potential response performance, which gives credit to the regulation of both the polymerization duration and the Au^3+^ concentration of the electrolyte. This suggests that the findings in this work not only improve the performance of the PPy-Au-NS ISE in this work, but also benefit the further performance improvement of other NS ISEs.

### 3.5. Long-Term Stability, Redox Sensitivity, and Ion Selectivity of the PPy-Au-NS ISE

**Figure 14:**
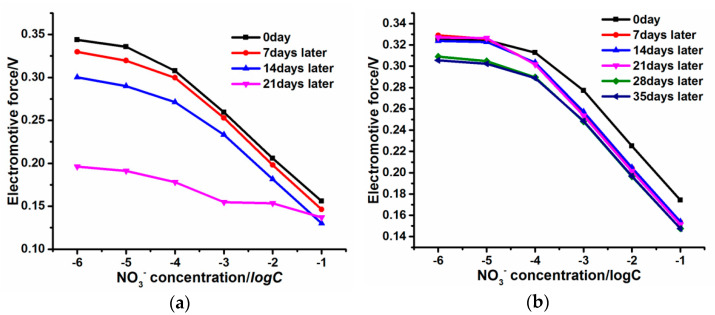

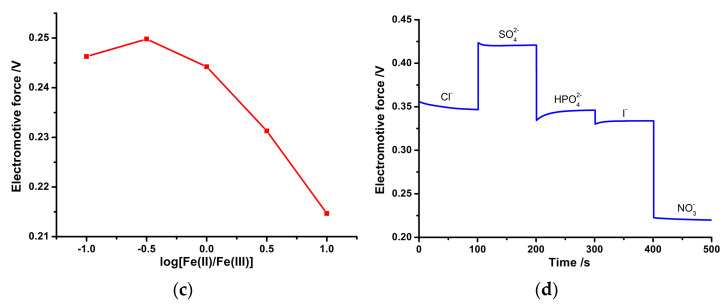
EMF dependence on the logarithm of the activity of PPy -NS ISE (**a**) and PPy-Au-NS ISE (**b**) days later. Redox sensitivity (**c**) and ion selectivity (**d**) of the PPy-Au-NS ISE. PPy (NO_3_^−^)-ISM was synthesized for polymerization duration 1800 s, while PPy-Au-NS ISE was synthesized at Au^3+^ concentration 2.5 mM of the electrolyte.

Figure 14a shows that after 7 days and 14 days, the EMFs corresponding to different NO_3_^−^ concentrations of the PPy-NS ISE for polymerization duration 1800 s reduce moderately. However, 21 days later, the EMFs decrease extremely, that is, the reliable working life of the PPy-NS ISE is about 2 weeks. For the PPy-Au-NS ISE at Au^3+^ concentration 2.5 mM of the electrolyte (Figure 14b), after 35 days, the EMFs corresponding to different NO_3_^−^ concentrations decrease slightly, and the Nernstian slope still remains high (above 50 mV/dec); this indicates that the PPy(NO_3_^−^)-ISM and Au solid contact layer still stably adhere with the surface of the GCE, and the reliable working life of the PPy-Au-NS ISE is longer than 5 weeks. These results are attributed to the fact that the hydrophobic Au solid contact layer avoids the formation of an aqueous layer in the PPy-Au-NS ISE. Moreover, Figure 14c reveals that the PPy-Au-NS ISE has a moderate redox sensitivity of 23.6 ± 3.5 mV/dec. This means that the PPy(NO_3_^−^)-ISM is a redox-sensitive electronic conductor [9,37,38], and redox buffer definitely affects the potentiometric response of the PPy-Au-NS ISE. This indicates that the PPy-Au-NS ISE is more suitable for NO_3_^−^ detection without redox interference. Figure 14d shows the different potential responses of the PPy-Au-NS ISE corresponding to various ion solutions. Accordingly, the selectivity coefficients of Cl^−^, SO_4_^2−^, HPO_4_^2−^, and I^−^ were determined to be −2.3, −3.7, −2.3, and −2.1, respectively. This suggests that the as-prepared PPy-Au-NS ISE is of high ion selectivity. These findings indicate that the PPy-Au-NS ISE, exhibiting prominent long-term stability and excellent ion selectivity, can be employed as an effective working electrode for NO_3_^−^ electrochemical detection without redox interference.

## 4. Conclusions

In this paper, PPy-Au-NS ISEs were fabricated with polymerization duration and the Au^3+^ concentration of the electrolyte. It was found that, for polymerization duration of 1800 s, PPy(NO_3_^−^)-ISM is the roughest and most hydrophilic, which leads to the largest solid–liquid contact surface area for NO_3_^−^ ion adsorption and electron generation, and subsequently gives rise to the optimal NO_3_^−^ potential response. Furthermore, at Au^3+^ concentration of the electrolyte 2.5 mM, the Au solid contact layer with sufficient hills and adequate pits is the most hydrophobic, prevents the formation of the aqueous layer in the PPy-Au-NS ISE, and further leads to optimal NO_3_^−^ potential response performance, that is, a Nernstian slope of 54.0 mV/dec, LOD of 1.1 × 10^−4^ M, rapid average response time of less than 1.9 s, and long-term stability longer than 5 weeks. These results benefit not only the performance improvements of the NS ISEs, but also the optimization of other all-solid ion-selective electrodes.

## Figures and Tables

**Table 1:** Comparison of nitrate-selective all-solid-state electrodes.

ISM	Solid Contact Layer	Nernstian Slope (mV/dec)	LOD (M)	Average Response Time (s)	Ref.
PPy(NO_3_^−^)	Au	54	10^−4^	<1.9	This work
MTDDANO_3_	CRGNO	57.9	10^−4^	<10	[13]
PPy(NO_3_^−^)	GR	56	10^−5^	<10	[18]
TDANO_3_	POT	53	10^−5^	\	[23]
TDANO_3_	Poly(aniline)	56	10^−5^	\	[23]
PPy(NO_3_^−^)	\	58	3 × 10^−5^	60	[35]
I–PVC–TBAB–DBP	\	57	4 × 10^−5^	<20	[36]

MTDDANO_3_, methyltridodecylammonium nitrate; TDANO_3_, tetradecylammonium nitrate; GR, graphene; CRGNO, chemically reduced graphene oxide.

## Data Availability

The data that support the findings of this study are available from the corresponding author, W. X. Jing, upon reasonable request.
